# Voxelwise characterization of noise for a clinical photon-counting CT scanner with a model-based iterative reconstruction algorithm

**DOI:** 10.1186/s41747-024-00541-2

**Published:** 2025-01-02

**Authors:** Luigi Masturzo, Patrizio Barca, Luca De Masi, Daniela Marfisi, Antonio Traino, Filippo Cademartiri, Marco Giannelli

**Affiliations:** 1 Unit of Medical Physics, Pisa University Hospital “Azienda Ospedaliero-Universitaria Pisana”, Pisa, Italy; 2 Siemens Healthcare s.r.l., Milano, Italy; 3 Medical Physics Department, Udine University Hospital “Azienda Sanitaria Universitaria Friuli Centrale”, Udine, Italy; 4 Department of Radiology, Fondazione Monasterio, Pisa, Italy

**Keywords:** Algorithms, Image processing (computer-assisted), Radiation exposure, Tomography (x-ray computed), Tomography scanners (x-ray computed)

## Abstract

**Background:**

Photon-counting detector (PCD) technology has the potential to reduce noise in computed tomography (CT). This study aimed to carry out a voxelwise noise characterization for a clinical PCD-CT scanner with a model-based iterative reconstruction algorithm (QIR).

**Methods:**

Forty repeated axial acquisitions (tube voltage 120 kV, tube load 200 mAs, slice thickness 0.4 mm) of a homogeneous water phantom and CTP404 module (Catphan-504) were performed. Water phantom acquisitions were also performed on a conventional energy-integrating detector (EID) scanner with a sinogram/image-based iterative reconstruction algorithm, using similar acquisition/reconstruction parameters. For smooth/sharp kernels, filtered back projection (FBP)- and iterative-reconstructed images were obtained. Noise maps, non-uniformity index (NUI) of noise maps, image noise histograms, and noise power spectrum (NPS) curves were computed.

**Results:**

For FBP-reconstructed images of water phantom, mean noise was (smooth/sharp kernel) 11.7 HU/51.1 HU and 18.3 HU/80.1 HU for PCD-scanner and EID-scanner, respectively, with NUI values for PCD-scanner less than half those for EID-scanner. Percentage noise reduction increased with increasing iterative power, up to (smooth/sharp kernel) 57.7%/72.5% and 56.3%/70.1% for PCD-scanner and EID-scanner, respectively. For PCD-scanner, FBP- and QIR-reconstructed images featured an almost Gaussian distribution of noise values, whose shape did not appreciably vary with iterative power. Noise maps of CTP404 module showed increased NUI values with increasing iterative power, up to (smooth/sharp kernel) 15.7%/9.2%. QIR-reconstructed images showed limited low-frequency shift of NPS peak frequency.

**Conclusion:**

PCD-CT allowed appreciably reducing image noise while improving its spatial uniformity. QIR algorithm decreases image noise without modifying its histogram distribution shape, and partly preserving noise texture.

**Relevance statement:**

This phantom study corroborates the capability of photon-counting detector technology in appreciably reducing CT imaging noise and improving spatial uniformity of noise values, yielding a potential reduction of radiation exposure, though this needs to be assessed in more detail.

**Key Points:**

- First voxelwise characterization of noise for a clinical CT scanner with photon-counting detector technology.
- Photon-counting detector technology has the capability to appreciably reduce CT imaging noise and improve spatial uniformity of noise values.
- In photon-counting CT, a model-based iterative reconstruction algorithm (QIR) allows decreasing effectively image noise.
- This is done without modifying noise histogram distribution shape, while limiting the low-frequency shift of noise power spectrum peak frequency.

**Graphical Abstract:**

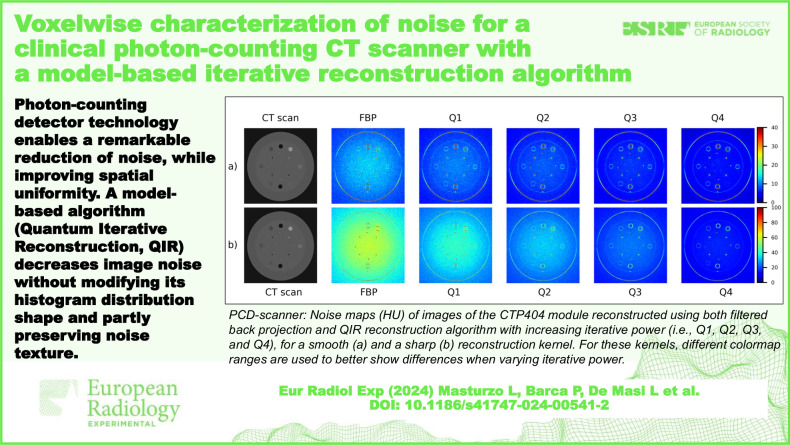

## Background

Given its potential advantages, computed tomography (CT) with photon-counting detector (PCD) technology represents a promising diagnostic tool for several clinical applications [1–3]. Specifically, the use of PCDs, as compared to conventional energy-integrating detectors (EIDs), might allow suppressing electronic noise, improving spatial resolution, reducing radiation exposure, and enhancing contrast-to-noise ratio for imaging with contrast media [4–6]. Moreover, due to the peculiarity of revealing each single photon and its energy, PCD technology yields intrinsically spectral CT imaging, with the capability of reconstructing virtual-monoenergetic images or images with specific energy ranges by using different energy thresholds [7–9].

Progressive advancements in detector technology have resulted in the first whole-body commercial photon-counting CT scanner (NAEOTOM Alpha, Siemens Healthineers, Forchheim, Germany) being cleared by the Food and Drug Administration for clinical use in late 2021, opening the possibility to effectively validate this new imaging modality and optimize its use for different patient real-world examinations [10]. While a limited number of preliminary clinical studies have shown encouraging results [4, 6, 11–20], further tailored technical as well as clinical studies are needed to better assess the actual impact of photon-counting CT for various applications. So far, only few previous studies have characterized the performances of preclinical [5, 21–25] or clinical [4, 6, 26–32] photon-counting CT scanners in terms of image quality for different settings.

All the previous studies have shown potential advantages of PCD-CT systems in terms of reduced image noise as compared to scanners with EIDs. It should be noted that, for noise estimation, they have used only a conventional region of interest (ROI)-based approach, while a rigorous voxel-by-voxel assessment through several repeated measurements can provide a more comprehensive and exhaustive characterization [33–35]. Therefore, the aim of this phantom study was to carry out a voxelwise characterization of noise properties for the only currently available clinical PCD-CT scanner, equipped with a novel model-based iterative reconstruction algorithm.

## Methods

### Phantoms, scanners, acquisitions, and image reconstructions

For image acquisitions, two different phantoms were employed: a standard homogeneous water phantom (diameter 20 cm) supplied by the scanners’ manufacturer and the CTP404 module (with different contrast objects) of the Catphan-504 phantom (The Phantom Laboratory, New York, NY, USA).

In the first part of our study, we planned and carried out data acquisition of both phantoms on a PCD-CT scanner NAEOTOM Alpha (Siemens Healthineers, Forchheim, Germany) using typical acquisition and reconstruction parameters (Table 1), with the fixed collimation (144 × 0.4 mm) of the standard acquisition modality. Given that PCD technology features a reduction of slice thickness in CT imaging, which is of paramount importance for various clinical applications such as cardiac and cardiovascular examinations [36, 37], we employed the minimum slice thickness of 0.4 mm, to exploit the potential of this technology. Each phantom acquisition was consecutively repeated 40 times, without table movement. Subsequently, in order to provide a more comprehensive evaluation and contextualization of the noise performances observed for PCD-CT imaging, water phantom acquisitions were performed also on a CT scanner Definition FLASH (Siemens Healthineers, Forchheim, Germany) with conventional EIDs, using a protocol similar to that used for the PCD-scanner (see Table 1). In particular, applying the same criterion adopted for the PCD-scanner, the maximum collimation (64 × 0.6 mm) and the minimum slice thickness (0.6 mm) available on the EID-scanner were adopted.

**Table 1:** Acquisition and reconstruction parameters for PCD-scanner and EID-scanner

	PCD-scanner	EID-scanner
Scan mode	Axial	Axial
Tube voltage (kV)	120	120
Tube load (mAs)	200	200
Rotation time (s)	0.5	0.5
Matrix size	512 × 512	512 × 512
Field of view (mm × mm)	220 × 220	220 × 220
Collimation (mm)	144 × 0.4	64 × 0.6
Slice thickness (mm)	0.4	0.6

*EID* Energy-integrating detector, *PCD* Photon-counting detector

For the PCD-scanner, images were obtained using T3D modality (*i.e*., considering only the detected photons with energy higher than 20 keV) [38]. Images were reconstructed using filtered back projection (FBP), as well as the model-based Quantum iterative reconstruction (QIR) algorithm with increasing iterative power (Q1, Q2, Q3, Q4) [28, 29, 39]. Moreover, for each reconstruction algorithm, two different reconstruction kernels (smooth and sharp) were adopted: Br40 and Br60. For the EID-scanner, images were reconstructed through FBP, as well as through the sinogram affirmed iterative reconstruction (SAFIRE) algorithm with increasing iterative power (S1, S2, S3, S4, S5) [40]. The employed reconstruction kernels were B40 (FBP and iterative reconstructions) and B60 (FBP reconstruction), which are equivalent to Br40 and Br60, respectively. Given that B60 reconstruction kernel was not available for iterative reconstructions with SAFIRE, the I50 kernel (slightly smoother than B60) was adopted.

### Image analysis

Noise maps were obtained on a voxel-by-voxel basis for the slice at the central level of acquired slab, computing the standard deviation of CT numbers across the 40 repeated acquisitions. Then, maps of the percentage difference between noise of FBP-reconstructed images and noise of images reconstructed through iterative algorithm with different iterative powers were computed voxelwise.

For CT imaging of the homogeneous water phantom, the noise level was estimated as mean ± standard deviation value of noise maps within a central circular ROI of 1 cm diameter. For CT imaging of the CTP404 module, the noise value of each contrast object was estimated as mean ± standard deviation of noise values within a circular ROI of 1 cm diameter placed in the center of the contrast object. To assess spatial non-uniformity of noise, the non-uniformity index (NUI), as proposed by the American Association of Physicists in Medicine Task Group 233 report, was employed [33, 34]. Specifically, for both phantoms, a large central ROI (diameter 15 cm) was divided into k = 249 small ROIs (size 8 mm × 8 mm). Then, for each noise map, NUI was calculated as:

1 $$NUI=\frac{100}{ < m > }\sqrt{\frac{1}{k-1}{\sum}_{i=1}^{k}{({m}_{i}- < m > )}^{2}}$$

where m_i_ and <m> are the average of noise values within the i-th ROI and the average of all m_i_ values, respectively. Furthermore, for water phantom acquisitions, histograms of image noise values (within a central ROI of 15 cm diameter) were obtained and characterized in terms of various descriptors such as median, interquartile range, skewness, and kurtosis (*i.e*., the fourth moment of the distribution minus 3).

Noise texture properties were assessed by performing noise power spectrum (NPS) analysis. In particular, we estimated the three-dimensional NPS, which is suitable for CT imaging due to its inherent multidimensional nature [41], as follows [42]:

2 $${NPS}({f}_{x},\,{f}_{y},\,{f}_{z})=\frac{1}{2}\left(\frac{\Delta x \, {{\Delta }}{\Delta y \, {{\Delta }}}{\Delta z}}{{N}_{x}{N}_{y}{N}_{z}}\right)* \left < \ {\left|{FFT}\left({VO}{I}_{{noise}\left(x,y,z\right)}\right)\right|}^{2}\right > $$

where (f_x_, f_y_, f_z_), (Δx, Δy, Δz), and (N_x_, N_y_, N_z_) represent the spatial frequencies along the main orthogonal directions (x, y, z), voxel sizes, and number of voxels along each direction, respectively; FFT and VOI_noise(x,y,z)_ are the three-dimensional fast Fourier transform and local values of an “only-noise” volume of interest (VOI), respectively; “< >” indicates the ensemble average across multiple calculations performed on several VOIs. The “only-noise” volumes were obtained by subtracting one set of images from the remaining 39 sets with identical scanning/reconstruction parameters (yielding the inclusion of the 1/2 factor in the NPS formula). For each image set, six different contiguous regions (with 5.4 mm length along *z* direction) of the water phantom were selected, avoiding the extremities to prevent edge effects. Then, four VOIs (composed of 128 × 128 voxels and not superimposed) were considered on each region, resulting in a total ensemble of 24 parallelepiped VOIs. After calculating the three-dimensional NPS and selecting the plane with f_z_ = 0, 100 different radial profiles (with uniform angular sampling) were averaged to obtain a representative radial NPS [33, 41]. The final NPS was obtained by averaging the radial NPS calculated over the 39 sets of images.

All image analyses were performed by using the Python software package (Python 3.10.8) [43].

## Results

**Fig. 1:**
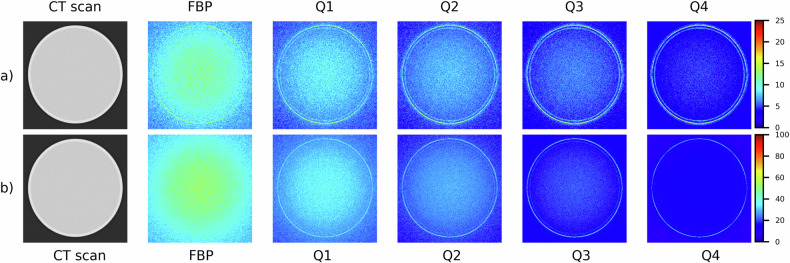
Photon-counting detector computed tomography scanner: noise (HU) maps of CT image of the water phantom reconstructed using both FBP and QIR algorithm with increasing iterative power (*i.e*., Q1, Q2, Q3, and Q4), for the smoother (**a**) and sharper (**b**) reconstruction kernel. For these reconstruction kernels, different colormap ranges are used to better show differences when varying iterative power. FBP, Filtered back projection; QIR, Quantum iterative reconstruction

**Fig. 2:**
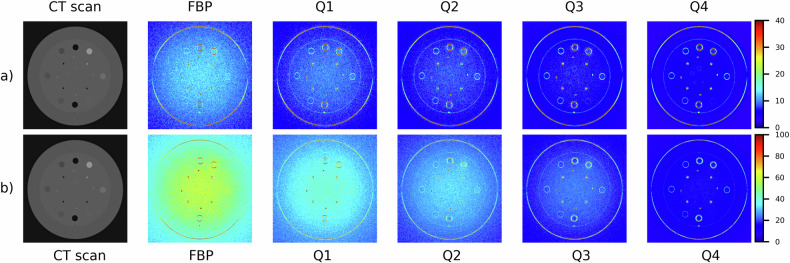
Photon-counting detector computed tomography scanner: noise (HU) maps of CT image of the CTP404 module reconstructed using both FBP and QIR algorithm with increasing iterative power (*i.e*., Q1, Q2, Q3, and Q4), for the smoother (**a**) and sharper (**b**) reconstruction kernel. For these reconstruction kernels, different colormap ranges are used to better show differences when varying iterative power*.* FBP, Filtered back projection; QIR, Quantum iterative reconstruction

**Fig. 3:**
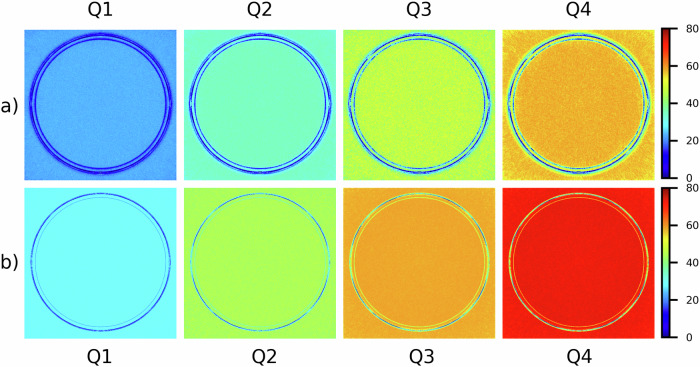
Photon-counting detector computed tomography scanner: for the water phantom, maps of the percentage difference (%) between noise of FBP-reconstructed image and noise of QIR-reconstructed images with different iterative powers (*i.e*., Q1, Q2, Q3, and Q4), using the smoother (**a**) and sharper (**b**) reconstruction kernel. FBP, Filtered back projection; QIR, Quantum iterative reconstruction

**Fig. 4:**
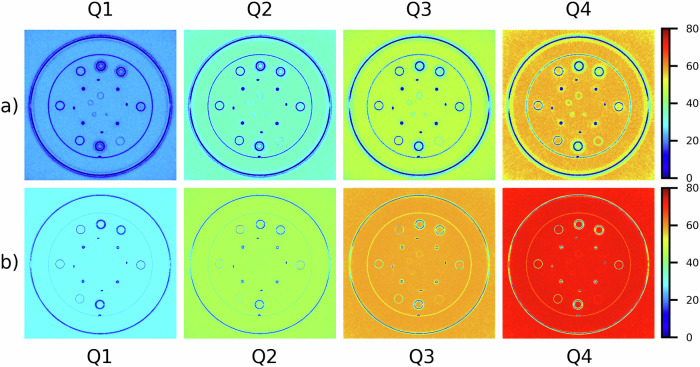
Photon-counting detector computed tomography scanner: for the CTP404 module, maps of the percentage difference (%) between noise of FBP-reconstructed image and noise of QIR-reconstructed images with different iterative powers (*i.e*., Q1, Q2, Q3, and Q4), using the smoother (**a**) and sharper (**b**) reconstruction kernel. FBP, Filtered back projection; QIR, Quantum iterative reconstruction

For the PCD-scanner, noise maps of acquisitions of water phantom and CTP404 module with different contrast objects are shown in Fig. 1 and Fig. 2, respectively. Moreover, maps of the percentage difference between noise of FBP-reconstructed images and noise of images reconstructed through QIR with different iterative powers are shown in Fig. 3 (homogeneous water phantom) and Fig. 4 (CTP404 module). The analogous maps for EID-scanner are reported in Supplementary Fig. S1 (noise maps) and Supplementary Fig. S2 (maps of the percentage difference between noise of FBP-reconstructed images and noise of SAFIRE-reconstructed images).

**Fig. 5:**
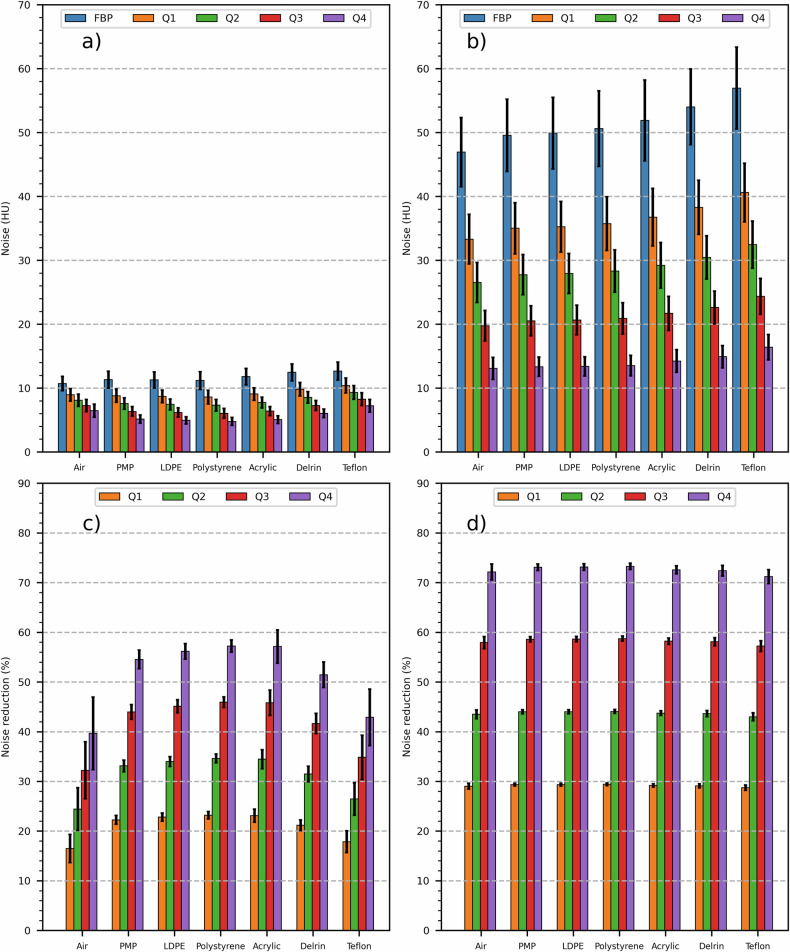
Photon-counting detector computed tomography scanner: CTP404 phantom module with different contrast objects (*i.e*., air, PMP, LDPE, polystyrene, acrylic, Delrin, Teflon): noise (**a**, **b**) and percentage noise reduction (**c**, **d**) values (mean ± standard deviation within a 1 cm diameter region of interest placed in each contrast object), for FBP and QIR algorithm with increasing power (*i.e*., Q1, Q2, Q3, and Q4), as well as for the smoother (**a**, **c**) and sharper (**b**, **d**) kernel. FBP, Filtered back projection; LDPE, Low-density polyethylene; PMP, Polymethylpentene; QIR, Quantum iterative reconstruction

For all acquisitions of the homogeneous water phantom (on both scanners) and reconstructions, noise values are reported in detail in Table 2. Notably, for the homogeneous water phantom acquired on PCD-scanner and EID-scanner, noise values in FBP-reconstructed images were (smoother/sharper kernel) 11.7 HU/51.1 HU and 18.3 HU/80.1 HU, respectively. Furthermore, when compared with FBP, QIR and SAFIRE iterative reconstruction algorithm allowed a substantial and similar reduction of noise with increasing power, up to (smoother/sharper kernel) 57.7%/72.5% and 56.3%/70.1% for the maximum iterative power (*i.e*., Q4 and S5), respectively. For CT images of the CPT404 module with different contrast objects obtained through PCD-scanner, noise and percentage noise reduction values are reported in Fig. 5. For all reconstructed images except when using smoother kernel and QIR algorithm, noise values increased monotonically with increasing relative electron density of the contrast object (*i.e*., from air to Teflon insert). Moreover, when using smoother kernel, maximum percentage noise reduction was lower for higher contrast objects, with values of approximately 40% and 55% for air/Teflon and other contrast objects, respectively; conversely, maximum percentage of noise reduction was approximately 72% for all contrast objects when using sharper kernel.

**Table 2:** Computed tomography image noise and noise reduction values in a homogeneous water phantom

Scanner	Reconstruction kernel	Reconstruction algorithm	Noise (HU)	Noise reduction
PCD	Smoother	FBP	11.7 ± 1.3	
		Q1	9.0 ± 1.0	23.2 ± 1.3%
		Q2	7.6 ± 0.8	35.3 ± 0.7%
		Q3	6.3 ± 0.7	46.2 ± 0.8%
		Q4	5.0 ± 0.6	57.7 ± 0.6%
	Sharper	FBP	51.1 ± 5.9	
		Q1	36.2 ± 4.2	29.2 ± 1.4%
		Q2	28.2 ± 3.4	44.8 ± 1.3%
		Q3	21.4 ± 2.5	58.2 ± 0.6%
		Q4	14.0 ± 1.6	72.5 ± 0.8%
EID	Smoother	FBP	18.3 ± 2.1	
		S1	16.2 ± 1.9	11.4 ± 1.5%
		S2	14.1 ± 1.7	23.0 ± 1.4%
		S3	12.1 ± 1.4	33.9 ± 1.2%
		S4	10.0 ± 1.2	44.8 ± 0.8%
		S5	8.0 ± 1.0	56.3 ± 0.6%
	Sharper	FBP	80.1 ± 9.1	
		S1	38.1 ± 4.4	52.4 ± 1.6%
		S2	36.5 ± 4.1	54.4 ± 1.5%
		S3	35.0 ± 3.9	56.3 ± 1.3%
		S4	28.8 ± 3.4	64.0 ± 0.9%
		S5	23.9 ± 3.1	70.1 ± 0.8%

Different reconstruction kernels (smoother/sharper) as well as FBP and iterative reconstruction algorithms (QIR/SAFIRE) with increasing power for PCD-scanner (Q1, Q2, Q3, Q4) and for EID-scanner (S1, S2, S3, S4, S5) were used. Noise and noise reduction values are reported as mean ± standard deviation within a central region of interest of 1 cm diameter

*EID* Energy-integrating detector, *FBP* Filtered back projection, *PCD* Photon-counting detector, *QIR* Quantum iterative reconstruction, *SAFIRE* Sinogram affirmed iterative reconstruction

**Fig. 6:**
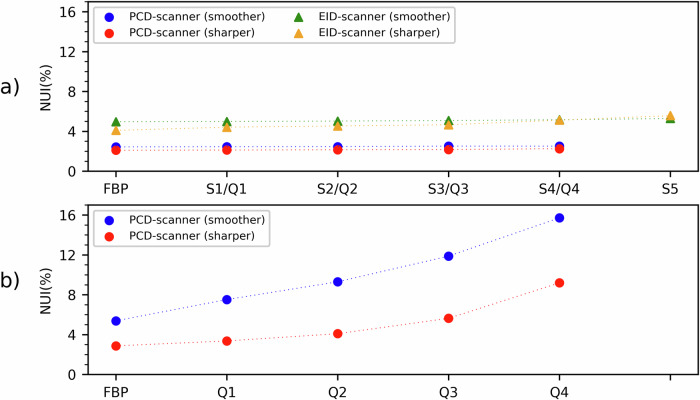
For homogeneous water phantom (**a**) and CTP404 module (**b**), NUI values of noise maps of computed tomography images acquired on PCD-scanner and EID-scanner, reconstructed using different kernels and both FBP and iterative algorithms with increasing power (*i.e*., Q1/Q2/Q3/Q4 and S1/S2/S3/S4/S5 for PCD-scanner and EID-scanner, respectively). EID, Energy-integrating detector; FBP, Filtered back projection; NUI, Non-uniformity index; PCD, Photon-counting detector

NUI results for noise maps are shown in Fig. 6. When considering the homogeneous water phantom (Fig. 6a), NUI values were approximately 2.5% and 5.3% for PCD-scanner and EID-scanner, respectively, with minimal dependence on reconstruction kernel and iterative reconstruction power. On the other hand, when considering the CTP404 module with different contrast objects acquired on PCD-scanner (Fig. 6b), NUI values for noise of FBP-reconstructed images were (smoother/sharper kernel) 5.4%/2.9%. Moreover, NUI values increased nonlinearly with increasing iterative reconstruction power, up to (smoother/sharper kernel) 15.7%/9.2% for maximum iterative reconstruction power (*i.e*., Q4).

For homogeneous water phantom acquisitions, values of image noise histogram descriptors (median, interquartile range, kurtosis, and skewness) are reported in Table 3. In this regard, image noise distribution was more similar to a Gaussian distribution (*i.e*., skewness and kurtosis values equal to zero) for FBP-reconstructed images obtained from PCD-scanner than EID-scanner. In addition, while skewness increased with increasing iterative power when using SAFIRE algorithm for EID-scanner, both skewness and kurtosis values did not appreciably vary when using QIR algorithm with increasing power for PCD-scanner.

**Table 3:** Computed tomography image noise histogram descriptors in a homogeneous water phantom

Scanner	Reconstruction kernel	Reconstruction algorithm	Median	IQR	Skewness	Kurtosis
PCD	Smoother	FBP	10.2	1.9	0.22	−0.02
		Q1	7.8	1.4	0.23	−0.02
		Q2	6.7	1.2	0.23	−0.02
		Q3	5.5	1.0	0.26	−0.02
		Q4	4.3	0.8	0.24	−0.01
	Sharper	FBP	45.6	8.1	0.21	−0.01
		Q1	32.2	5.7	0.21	−0.01
		Q2	25.6	4.6	0.23	0.01
		Q3	18.9	3.4	0.22	0.00
		Q4	12.4	2.2	0.23	0.01
EID	Smoother	FBP	14.0	3.5	0.43	−0.06
		S1	12.4	3.1	0.44	−0.05
		S2	10.7	2.7	0.44	−0.03
		S3	9.1	2.3	0.45	−0.01
		S4	7.5	1.9	0.48	0.07
		S5	5.9	1.5	0.53	0.22
	Sharper	FBP	63.9	14.4	0.36	−0.06
		S1	29.9	6.9	0.38	−0.05
		S2	28.4	6.6	0.36	−0.09
		S3	27.1	6.4	0.36	−0.10
		S4	21.7	5.5	0.39	−0.05
		S5	17.4	4.7	0.44	0.02

Two different reconstruction kernels (smoother/sharper) as well as FBP and iterative reconstruction algorithms (QIR/SAFIRE) with increasing power for PCD-scanner (Q1, Q2, Q3, Q4) and for EID-scanner (S1, S2, S3, S4, S5) were used

*EID* Energy-integrating detector, *FBP* Filtered back projection, *IQR* Interquartile range, *PCD* Photon-counting detector, *QIR* Quantum iterative reconstruction, *SAFIRE* Sinogram affirmed iterative reconstruction

NPS curves of PCD-scanner and EID-scanner are shown in Supplementary Fig. S3 (smoother reconstruction kernel) and Supplementary Fig. S4 (sharper reconstruction kernel). Additionally, peak frequency (f_p_) values of the NPS curves are reported in Table 4. When FBP-reconstructed images were considered, f_p_ values for PCD-scanner were lower (smoother kernel) or equal (sharper kernel) to those for EID-scanner. Moreover, the use of iterative reconstruction algorithms for both scanners resulted in a low-frequency shift of f_p_ (with respect to that of FBP-reconstructed images) with increasing iterative reconstruction power. In particular, only the maximum iterative reconstruction power resulted in an appreciable shift for PCD-scanner, and the maximum percentage low-frequency shift of f_p_ was similar between PCD-scanner and EID-scanner for smoother reconstruction kernel.

**Table 4:** Peak frequency of noise power spectrum curves (f_p_) of computed tomography images

Scanner	Reconstruction kernel	Reconstruction algorithm	f_p_ (mm^-1^)
PCD	Smoother	FBP	0.21
		Q1	0.21
		Q2	0.21
		Q3	0.21
		Q4	0.15
	Sharper	FBP	0.57
		Q1	0.57
		Q2	0.57
		Q3	0.55
		Q4	0.48
EID	Smoother	FBP	0.26
		S1	0.26
		S2	0.25
		S3	0.19
		S4	0.19
		S5	0.19
	Sharper	FBP	0.57
		S1	0.41
		S2	0.41
		S3	0.41
		S4	0.41
		S5	0.37

Two different reconstruction kernels (smoother/sharper), as well as FBP and iterative reconstruction algorithms (QIR/SAFIRE) with increasing power for PCD-scanner (Q1, Q2, Q3, Q4) and for EID-scanner (S1, S2, S3, S4, S5) were used

*EID* Energy-integrating detector, *FBP* Filtered back projection, *PCD* Photon-counting detector, *QIR* Quantum iterative reconstruction, *SAFIRE* Sinogram affirmed iterative reconstruction

## Discussion

Noise is a fundamental factor on which the performance of a CT scanner depends. While noise is usually estimated as the standard deviation of CT numbers within a relatively small region of interest, this approach would be conceptually appropriate only for ergodic (*i.e*., when time averages of properties equal ensemble averages, indicating that all accessible states are explored over time) and stationary imaging systems, which feature uncorrelated noise [35, 44]. Actually, due to image reconstruction and processing, CT image noise may present some degrees of correlation [41, 45, 46]. Moreover, this approach cannot provide information on the degree of spatial non-uniformity of noise [33]. Therefore, a more rigorous characterization of noise properties should be performed by computing voxelwise noise maps through several repeated acquisitions [33, 44, 47]. Notably, these issues might be of particular relevance for iterative reconstruction algorithms, given their inherent nonlinearity and nonstationarity properties. To the best of our knowledge, this is the first study that characterizes voxelwise noise properties for the only clinical scanner that currently implements PCD technology and a dedicated model-based iterative reconstruction algorithm.

Our main results suggest that PCD technology in CT imaging has potential advantages over conventional EID technology for reducing both noise and its spatial variability. When considering FBP-reconstructed images of the homogeneous water phantom, PCD-scanner presented a relevant noise reduction of 36% (smoother kernel) and 36.2% (shaper kernel) with respect to EID-scanner. This effect is even more relevant when considering that slice thickness was smaller for PCD-scanner (0.4 mm) than EID-scanner (0.6 mm). In this way, our results show that PCD technology has the potential to effectively reduce image noise while reducing the minimum slice thickness when compared to EID technology. On the other hand, the appreciable noise reduction capability featured by PCD-scanner is expected to increase further when comparing data with the same slice thickness. Based on results of a recent study by Bhattarai et al [27], the percentages of noise reduction that we observed might be higher for acquisitions with lower radiation exposure and phantoms with greater size. Furthermore, while NUI values of noise of FBP-reconstructed images of the homogeneous water phantom were relatively small for both scanners, NUI values for PCD-scanner were less than half those for EID-scanner, highlighting a potential of PCD technology in reducing spatial variability in local noise values as well.

We evaluated noise reduction performances of the new model-based QIR algorithm, associated with PCD technology. For homogeneous water phantom, QIR-reconstructed images showed an increased percentage of noise reduction (with respect to FBP-reconstructed images) with increasing iterative power, up to 57.7% (smoother kernel) and 72.5% (sharper kernel) for Q4 iterative power. These percentages of noise reduction are similar to those observed for SAFIRE-reconstructed images acquired on EID-scanner (Table 2) or when using other iterative reconstruction algorithms implemented on conventional scanners with EID technology [35, 48–54]. On the other hand, we point out that, unlike CT imaging with EID-scanner and SAFIRE iterative reconstruction algorithm, noise distribution of FBP- and QIR-reconstructed images for PCD-scanner was almost Gaussian, and its shape (in terms of skewness and kurtosis) did not vary considerably with iterative reconstruction power.

When considering CTP404 module with different contrast objects and both reconstruction kernels (smoother/sharper), NUI values of noise maps were higher for QIR-reconstructed images than FBP-reconstructed images, and they increased with increasing iterative power, with higher NUI values for smoother than sharper reconstruction kernel; in particular, NUI values for noise maps of QIR-reconstructed images with maximum iterative power (*i.e*., Q4) were approximately threefold that of noise maps for FBP-reconstructed images. This is likely to derive from a noise reduction capability of QIR algorithm that does not seem so effective at the boundary between different media (Figs. 2 and 4). While such an effect has been reported for other iterative reconstruction algorithms [35, 55–57], no previous study has observed it for CT imaging with QIR and PCD technology. As submitted by Solomon and Samei [56], given that iterative reconstruction algorithms aim at reducing noise while possibly preserving the fidelity of fine details, this necessitates a conservative approach to noise reduction for voxels near edges, and hence noise is not reduced as much near edges or structures (*i.e*., “edge” effect).

NPS results of FBP-reconstructed images using smoother kernel showed lower f_p_ values for PCD-scanner than EID-scanner, while both scanners exhibited similar f_p_ values for the sharper reconstruction kernel. QIR-reconstructed images featured a low-frequency shift of f_p_ with increasing iterative power, as revealed for SAFIRE-reconstructed images and reported for other iterative reconstruction algorithms [58, 59]. In this regard, while SAFIRE-reconstructed images presented an appreciable low-frequency shift of f_p_ already at middle iterative reconstruction powers (*i.e*., S2/S3), QIR-reconstructed images showed relevant low-frequency shift of f_p_ only at maximum iterative reconstruction power (*i.e*., Q4) for both smoother and sharper reconstruction kernels. This suggests the potential of QIR algorithm in preserving the original noise texture of FBP-reconstructed images.

We note that only a limited number of previous studies have assessed the image quality of PCD-CT technology. For instance, Yu et al [24] have evaluated noise performances of a research PCD-CT system adopting an anthropomorphic thorax phantom. Using a simplified model which accounts for quantum and electronic component of noise, they have highlighted the potential of PCDs in suppressing the electronic component with respect to conventional EIDs. Rajendran et al [5] have assessed image quality of CT images obtained on a PCD-CT prototype system. In particular, image uniformity, CT number accuracy, image noise, noise power spectrum, and spatial resolution were evaluated and compared to those of a conventional EID-CT scanner. Higher spatial resolution and lower image noise were associated with PCD-CT imaging, while no substantial changes in NPS shape (as compared to FBP-reconstructed images) were observed when iterative reconstructions were carried out for PCD-CT, unlike that typically observed for energy-integrating systems. Another recent study by Rajendran et al [4] has assessed the performance of the NAEOTOM Alpha PCD-CT scanner in terms of CT number accuracy and uniformity, low- and high-contrast spatial resolution, image noise, and NPS. In agreement with a previous study [5], when compared to an energy-integrating CT scanner, remarkable improvement in spatial resolution and lower image noise can be obtained by this type of scanner. A study conducted by Sartoretti et al [28] has focused on the employment of a new iterative reconstruction algorithm implemented on the NAEOTOM Alpha PCD-CT scanner. With respect to FBP, they have reported a noise magnitude reduction of up to 46% using the maximum level of the iterative reconstruction. Additionally, as compared to FBP-reconstructed images, they have found a contrast-to-noise ratio increment of approximately 70% in the liver region of patient images retrospectively reconstructed through the iterative algorithm [28]. In a recent phantom study, Bhattarai et al [27] performed a task-based assessment of the performances of the NAEOTOM Alpha PCD-CT scanner as compared to an energy-integrating CT scanner, for different phantom sizes and reconstruction kernels; the photon-counting scanner offered better image quality than the energy-integrating scanner for each phantom size, depending on the reconstruction kernel. Therefore, our phantom study further explores and characterizes the potential of CT imaging with PCD technology. In particular, we have shown that PCD-scanner with model-based QIR algorithm features relevant and promising noise performances (in terms of overall noise level, spatial uniformity and distribution of noise values, NPS). Nonetheless, this does not necessarily imply an appreciable radiation exposure reduction in clinical applications, which can depend also on other factors, such as the CT scanner performance in terms of low contrast detectability, which hence should be possibly assessed through task-based approaches. Moreover, the actual radiation exposure reduction capability of CT imaging with PCD technology needs to be assessed in detail through tailored clinical studies for different specific applications.

We recognize some potential limitations of this preliminary study, which are mainly due to the necessity and complexity of carrying out many repeated CT acquisitions (for two phantoms and two scanners, as performed in our work) to obtain noise maps. First, we considered only a single tube load value. On the other hand, the use of multiple tube load levels, especially in the ultra-low radiation exposure regimen (where the PCD technology might further exploit its inherent potential), would be of practical interest. Second, only the standard scan mode has been considered for PCD-scanner. While this acquisition modality can be employed in several clinical applications, in further tailored studies it would be important to conduct a voxelwise assessment of noise properties by considering also the ultra-high resolution modality (useful for specific clinical examinations). Third, we performed acquisitions of a cylindrical water phantom and the Catphan-504 phantom, which are rather reproducible across different centers. However, acquisitions of an anthropomorphic phantom might be useful to better characterize spatial noise non-uniformity for some clinical applications.

In summary, the results of this technical study corroborate the capability of PCD technology in appreciably reducing CT imaging noise, with improved spatial uniformity of noise values, as compared to conventional EID technology. Moreover, the model-based QIR algorithm allows decreasing image noise considerably with respect to FBP without modifying the shape of image noise histogram distribution (almost Gaussian) even for high iterative powers, as well as limiting low-frequency shift of NPS peak frequency.

## Supplementary information

**Additional file 1:** **Figure S1** Computed tomography energy-integrating detector scanner: noise maps (HU) of image of the water phantom reconstructed using both FBP and SAFIRE iterative reconstruction algorithm with increasing iterative power (*i.e.*, S1, S2, S3, S4, and S5), for the smoother (**a**) and sharper (**b**) reconstruction kernel. For these reconstruction kernels, different colormap ranges are used to better show differences when varying iterative power. **Figure S2** Computed tomography energy-integrating detector scanner: for the water phantom, maps of the percentage difference (%) between noise of FBP-reconstructed image and noise of SAFIRE-reconstructed images with different iterative powers (*i.e.*, S1, S2, S3, S4, and S5), using the smoother (**a**) and sharper (**b**) reconstruction kernel. **Figure S3** NPS curves of computed tomography images acquired on PCD-scanner (**a**) and EID-scanner (**b**) using smoother kernel and both FBP and iterative algorithms with increasing power (*i.e.*, Q1/Q2/Q3/Q4 and S1/S2/S3/S4/S5 for PCD-scanner and EID-scanner, respectively). **Figure S4** NPS curves of computed tomography images acquired on PCD-scanner (**a**) and EID-scanner (**b**) using sharper kernel and both FBP and iterative algorithms with increasing power (*i.e.*, Q1/Q2/Q3/Q4 and S1/S2/S3/S4/S5 for PCD-scanner and EID-scanner, respectively).

## Data Availability

The dataset used and/or analyzed during the current study is available from the corresponding author upon reasonable request.

## Abbreviations

CT: Computed tomography
EID: Energy-integrating detector
FBP: Filtered back projection
f_p_: Noise power spectrum peak frequency
NPS: Noise power spectrum
NUI: Non-uniformity index
PCD: Photon-counting detector
QIR: Quantum iterative reconstruction
ROI: Region of interest
SAFIRE: Sinogram affirmed iterative reconstruction
VOI: Volume of interest
