# Supplementary material for: Voxelwise characterization of noise for a clinical photon-counting CT scanner with a model-based iterative reconstruction algorithm
Source: Eur Radiol Exp. 2025 Jan 2;9:2. doi: 10.1186/s41747-024-00541-2 (PMC11695565; doi:10.1186/s41747-024-00541-2)
Supplement: Supplementary file 1 — Additional file 1: Figure S1 Computed tomography energy-integrating detector scanner: noise maps (HU) of image of the water phantom reconstructed using both FBP and SAFIRE iterative reconstruction algorithm with increasing iterative power (i.e., S1, S2, S3, S4, and S5), for the smoother (a) and sharper (b) reconstruction kernel. For these reconstruction kernels, different colormap ranges are used to better show differences when varying iterative power. Figure S2 Computed tomography energy-integrating detector scanner: for the water phantom, maps of the percentage difference (%) between noise of FBP-reconstructed image and noise of SAFIRE-reconstructed images with different iterative powers (i.e., S1, S2, S3, S4, and S5), using the smoother (a) and sharper (b) reconstruction kernel. Figure S3 NPS curves of computed tomography images acquired on PCD-scanner (a) and EID-scanner (b) using smoother kernel and both FBP and iterative algorithms with increasing power (i.e., Q1/Q2/Q3/Q4 and S1/S2/S3/S4/S5 for PCD-scanner and EID-scanner, respectively). Figure S4 NPS curves of computed tomography images acquired on PCD-scanner (a) and EID-scanner (b) using sharper kernel and both FBP and iterative algorithms with increasing power (i.e., Q1/Q2/Q3/Q4 and S1/S2/S3/S4/S5 for PCD-scanner and EID-scanner, respectively). [file 41747_2024_541_MOESM1_ESM.pdf]

Voxelwise characterization of noise for a clinical photon-counting CT scanner with a model-based iterative reconstruction algorithm

ELECTRONIC SUPPLEMENTARY MATERIAL

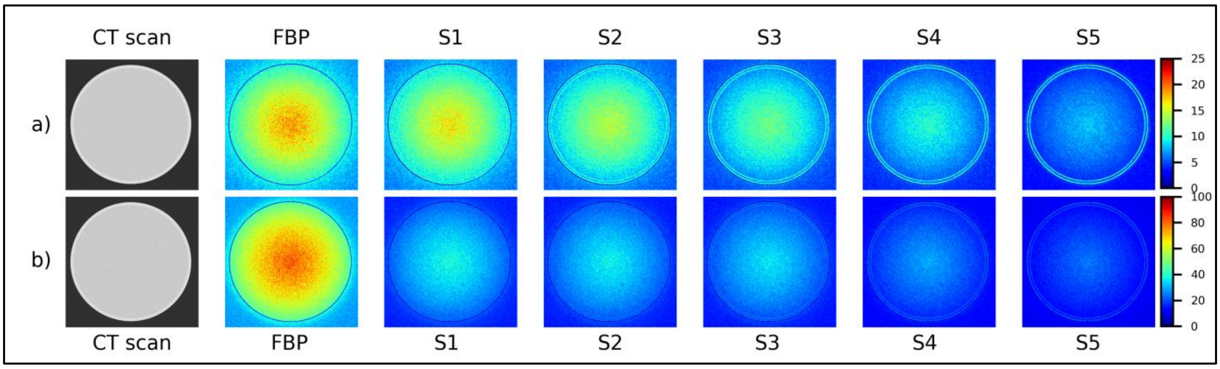

Figure S1 Computed tomography energy-integrating detector scanner: noise maps (HU) of image of the water phantom reconstructed using both FBP and SAFIRE iterative reconstruction algorithm with increasing iterative power (i.e., S1, S2, S3, S4, and S5), for the smoother (a) and sharper (b) reconstruction kernel. For these reconstruction kernels, different colormap ranges are used to better show differences when varying iterative power. FBP Filtered back projection, SAFIRE Sinogram affirmed iterative reconstruction.

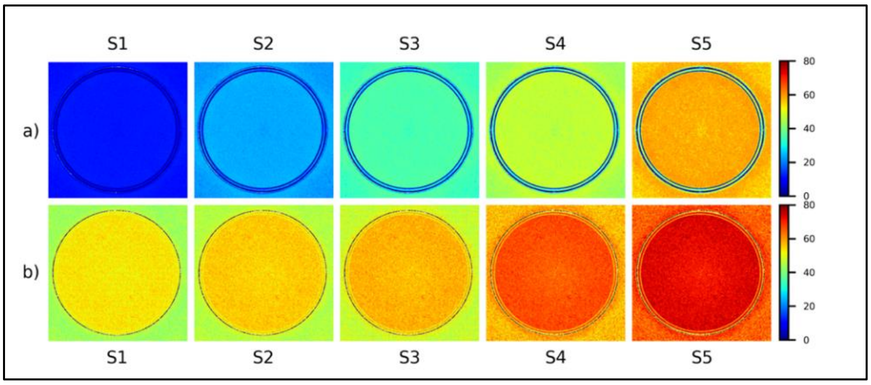

Figure S2 Computed tomography energy-integrating detector scanner: for the water phantom, maps of the percentage difference (%) between noise of FBP-reconstructed image and noise of SAFIRE-reconstructed images with different iterative powers (i.e., S1, S2, S3, S4, and S5), using the smoother (a) and sharper (b) reconstruction kernel. FBP Filtered back projection, SAFIRE Sinogram affirmed iterative reconstruction.

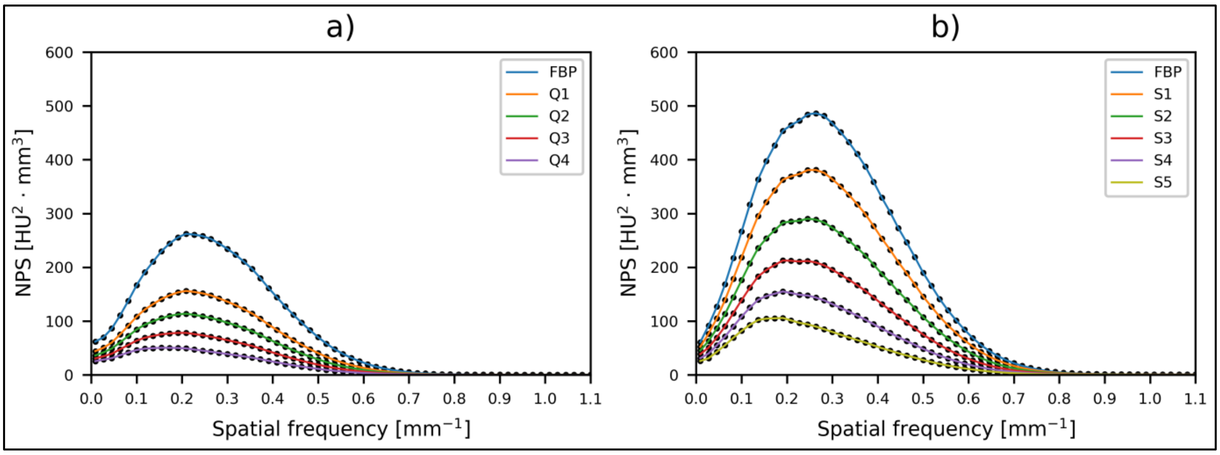

Figure S3 NPS curves of computed tomography images acquired on PCD-scanner (a) and EID-scanner (b) using smoother kernel and both FBP and iterative algorithms with increasing power (i.e., Q1/Q2/Q3/Q4 and S1/S2/S3/S4/S5 for PCD-scanner and EID-scanner, respectively). EID Energy-integrating detector, FBP Filtered back projection, NPS Noise power spectrum, PCD Photon-counting detector.

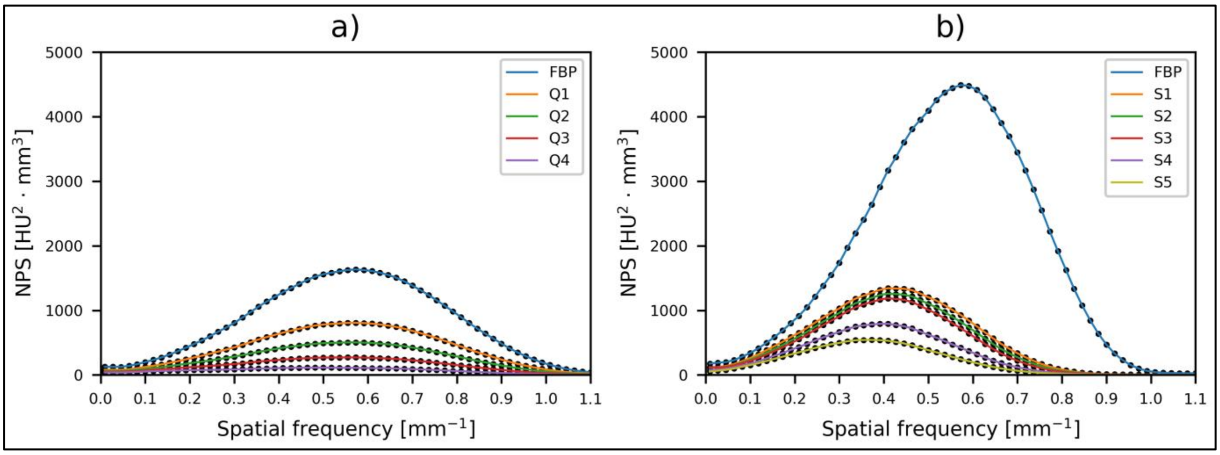

Figure S4 NPS curves of computed tomography images acquired on PCD-scanner (a) and EID-scanner (b) using sharper kernel and both FBP and iterative algorithms with increasing power (i.e., Q1/Q2/Q3/Q4 and S1/S2/S3/S4/S5 for PCD-scanner and EID-scanner, respectively). EID Energy-integrating detector, FBP Filtered back projection, NPS Noise power spectrum, PCD Photon-counting detector.
